# Multi-Modality Cardiac Imaging for Pericardial Diseases: A Contemporary Review

**DOI:** 10.31083/j.rcm2310336

**Published:** 2022-10-10

**Authors:** Tom Kai Ming Wang, Allan L Klein

**Affiliations:** ^1^Center for Diagnosis and Treatment of Pericardial Diseases, Heart, Vascular and Thoracic Institute, Cleveland Clinic, Cleveland, OH 44195, USA; ^2^Section of Cardiovascular Imaging, Heart, Vascular and Thoracic Institute, Cleveland Clinic, Cleveland, OH 44195, USA

**Keywords:** pericardium, echocardiography, computed tomography, magnetic resonance imaging, multi-modality cardiac imaging, pericarditis, pericardial effusion, constrictive pericarditis

## Abstract

Advances in multi-modality cardiac imaging have aided the evaluation, surveillance and treatment guidance of pericardial diseases, which have traditionally been a challenging group of conditions to manage. Although echocardiography remains the first-line imaging modality to assess the pericardium, both computed tomography (CT) and magnetic resonance imaging (MRI) have valuable complimentary roles. It is critical for clinicians to have a clear understanding of the utilities, advantages and disadvantages of these cardiac imaging modalities in pericardial pathologies. This contemporary review provides an update regarding the applications of multi-modality cardiac imaging in the evaluation of pericardial syndromes including acute/recurrent pericarditis, effusion/tamponade, constriction, masses and congenital anomalies.

## 1. Introduction

The human pericardium is an important structure that can present with a wide range of pathologies such as inflammation, effusion, constriction, masses and congenital anomalies, of varying time course and severity [1]. Given their potential adverse health burden, accurate diagnosis incorporating multi-modality cardiac imaging is critical to guide treatment decisions, with the goal to improve patient outcomes. The main pillars of cardiac imaging include echocardiography, computed tomography (CT) and magnetic resonance imaging (MRI), each with unique roles, strengths and limitations summarized in Table 1, and a scorecard of their utilities in Table 2 for various pericardial conditions [2]. Advances in multi-modality imaging alongside novel therapeutics has allowed the field of pericardial disease to grow in recent years. This review will outline the contemporary clinical applications of cardiac imaging modalities in pericardial clinical syndromes.

**Table 1.** **Advantages and disadvantages of cardiac imaging modalities for the evaluation of pericardial conditions**.

Imaging modality	Advantages	Disadvantages
Echocardiography	Portability	Operator dependent
	Availability	Body habitus dependent, can have limited views
	High temporal resolution	Lower spatial resolution
	Effusion, tamponade and constriction assessment	Lack tissue characterization
	Assess unstable patients	
	Intraprocedural guidance	
Computed tomography	Short study	Non-portable
	Spatial resolution	Ionizing radiation
	Detecting calcification	Contrast (avoid renal failure)
	Some tissue characterization	Motion artefact especially if non-gated or arrhythmia
	Extracardiac and vascular assessment	
	Pre-procedural planning	
Magnetic resonance imaging	Spatial resolution	Non-portable
	Tissue characterization	Lower availability
	Inflammation assessment	Longer study
	Chamber quantification (gold standard)	Contrast
	Extracardiac and vascular assessment	Breath-holding
		Claustrophobia
		Cardiac devices
		Detecting calcification

**Table 2.** **Scorecard and roles of multi-modality imaging in pericardial conditions**.

Pericardial disease	Echocardiography	Computed tomography	Magnetic resonance imaging
Acute pericarditis	*	*	***
	1	0	1
Recurrent pericarditis	*	*	***
	1	0	1
Pericardial effusion	***	**	*
	1	2	3
Pericardial tamponade	***	*	*
	1	2	0
Pericardial constriction	***	*	**
	1	3	2
Pericardial masses	*	**	***
	1	3	2
Congenital absence of pericardium	*	***	***
	0	1	1

*limited utility, **moderate utility, ***high utility/preferred option; 1 = first line test should be routinely performed for assessing this condition (can be more than one); 2 = second line test can be considered if there are uncertainties after first line test (can be more than one, which means one or more of the second line tests may be performed); 3 = third line test can be considered if there are still uncertainties after the first and second line tests were done, and 0 = test usually does not need to be performed.

## 2. Pericardial Anatomy and Function

Anatomically, the pericardium is made up of the visceral pericardium or inner serosa layer that adheres to the epicardium, as well as the relatively avascular parietal pericardium or outer fibrosa layer [3]. The visceral serosa layer contains a thin layer of mesothelial cells, while the parietal fibrosa layer contains an abundance of fibrous and some elastic tissues. Adipose fat tissue can be seen in the epicardial layer or adjacent to the pericardium outside of the pericardial space. A number of reflections of the visceral layer form recesses including the transverse and oblique sinuses, which can be visualized on cardiac imaging [4]. The pericardium surrounds the entire heart and the origin of the great vessels entering and existing the four heart chambers, where it is continuous with adventitia of these vessels. There is often adipose fat tissue in the pericardium is normally less than 2 mm thick, which is not visible by echocardiography unless it is abnormally thickened, but can often be seen on CT and MRI [2, 5].

Pericardial fluid lies in the potential physiological space between the visceral and parietal layers, normally up to 50 mL as the pericardial reserve volume [3]. Fluid pockets can also accumulate in the aforementioned sinuses and recesses. The fluid originates as plasma ultrafiltrate from pericardial capillaries and drains into the lymphatic system [2]. The pericardium is supplied by pericardiophrenic and musculophrenic arteries from the internal thoracic artery, and blood drains via the pericardiophrenic veins to the internal thoracic veins.

The main functions of the pericardium are to provide a mechanical and immunological barrier between the heart to protect it from trauma and infections respectively from the lungs, pleura, chest wall and other mediastinal structures [2]. It limits the over-distension of cardiac chambers to maintain their geometry and valve annulus during normal cardiac motion and volume overload states, while allowing for atria and ventricular interaction and coupling. The pericardial fluid contains prostaglandins from endothelial and mesothelial cells and affect cardiac reflexes and coronary tone, in addition being a lubricant to facilitate smooth cardiac motion [6].

## 3. Pericarditis (Acute, Recurrent and Chronic)

### 3.1 Echocardiography

**Fig. 1.**
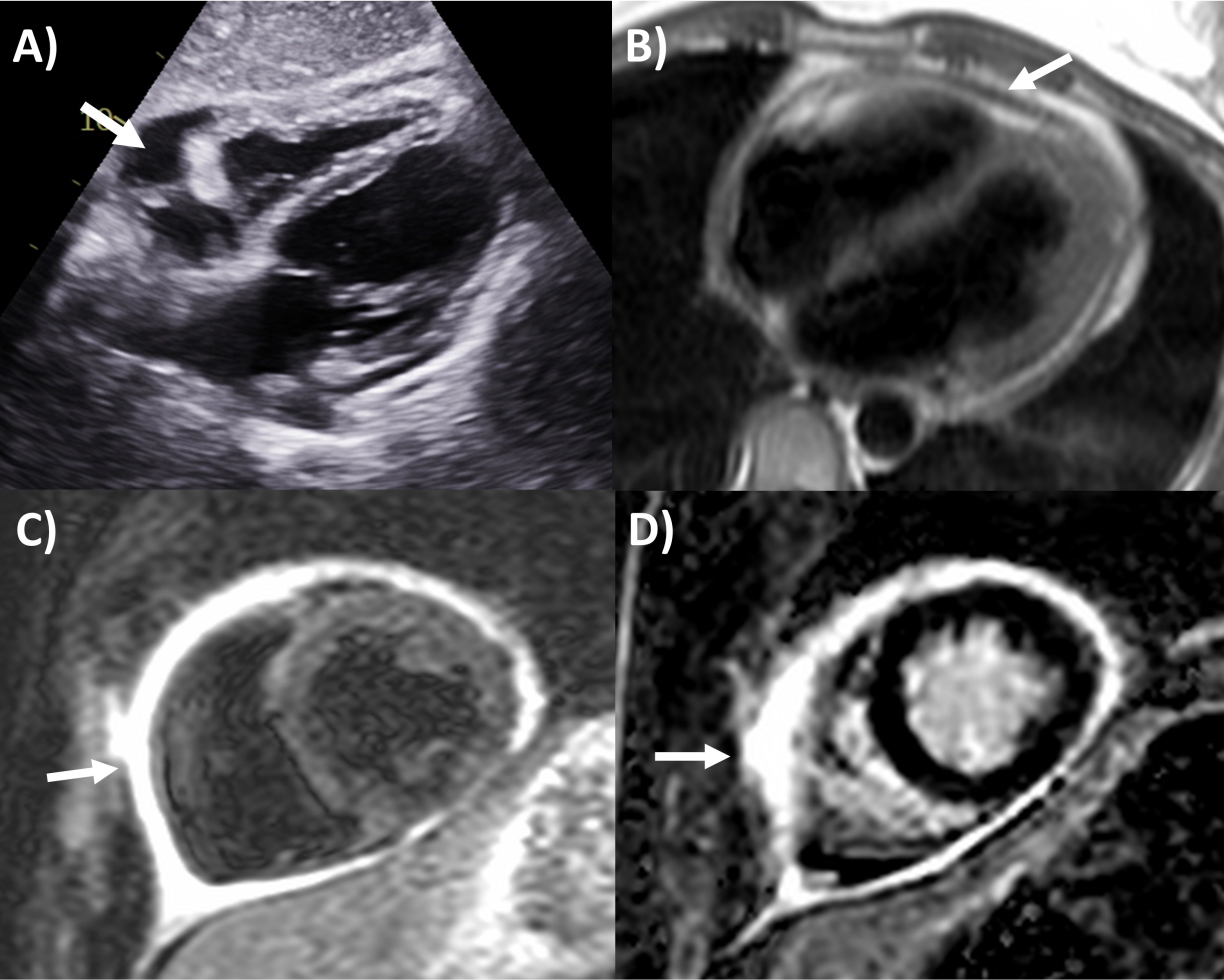
**Multi-modality imaging features of acute pericarditis case**. (A) Small pericardial effusion (arrow) subcostal view of echocardiography. (B) Pericardial thickening (arrow) on black-blood spin echo sequence of magnetic resonance imaging (MRI). (C) Severe circumferential increased pericardial signal indicating edema (arrow) on T2-weighted short tau inversion recovery imaging of MRI. (D) Severe circumferential pericardial enhancement indicating inflammation/fibrosis (arrow) on delayed gadolinium enhancement sequence of MRI.

Pericarditis is classified based on duration of the acute episode—acute for those with pericarditis for less than 4 weeks; incessant for ongoing pericarditis between at least 4–6 weeks but less than 3 months without remission, chronic for pericarditis lasting for over 3 months, and recurrent pericarditis refers to 2 or more episodes of acute pericarditis with at least a 4–6 week interval without symptoms [1]. Recurrent pericarditis events occur in 15–30% after the first episode of pericarditis, and in 50% after 2 or more episodes [7, 8, 9, 10]. The diagnostic criteria for pericarditis based on guidelines include at least 2 out of the 4 criteria of (1) pericarditis chest pain, (2) pericardial rub on physical examination, (3) new ECG changes (widespread ST-elevation or PR depression) and new or worsening pericardial effusion; while other supporting features include elevated inflammatory markers (such as C-reactive protein, sedimentation rate and white cell count), and imaging evidence of pericardial inflammation (especially by MRI, Fig. 1) [1]. The main etiology categories of pericarditis are idiopathic, infective (most commonly viral, but also bacterial including tuberculosis, fungal and parasitic), autoimmune, iatrogenic (such as post-cardiac surgery and interventions), neoplastic, metabolic and drug-related. Tuberculosis pericarditis is mainly prevalent in developing countries and rare in developed countries.

Based on this criteria, echocardiography is the first-line imaging modality recommended for all patients undergoing pericarditis evaluation, although it is often normal [1, 2]. Apart from its main role in identifying pericardial effusion, echocardiography can examine the presence of tamponade physiology present in approximately 3% of acute pericarditis, identify pericardial thickening, evaluate for the presence of regional wall motion abnormalities that may indicate concurrent myocardial involvement (myo-pericarditis) in approximately 5%, or look for alternative diagnoses like acute coronary syndrome or aortic dissection [2]. Echocardiography is also important during follow-up after initial pericarditis event for resolution of pericardial effusion if it had been present, along with signs of constrictive physiology, discussed in the later section.

### 3.2 Computed Tomography

In contrast to echocardiography and MRI, CT has a limited role for evaluating pericardial inflammation. Pericardial thickening which may enhance of iodinated contrast and the presence of pericardial effusion may be observed on chest CT when there is pericarditis [11]. CT is however usually ordered for evaluating alternative causes of chest symptoms such as coronary artery disease, acute aortic syndrome, pulmonary thromboembolism and other lung pathologies [12]. In chronic pericarditis, CT can identify calcifications that may indicate the presence of constriction that should be confirmed by other imaging modalities [2].

### 3.3 Magnetic Resonance Imaging

Perhaps the most important application of MRI in pericardial diseases is its ability to identify pericardial inflammation [2, 10]. The key features include pericardial thickening, best assessed on black-blood spin echo sequences; pericardial edema, assessed using T2-short tau inversion recovery (STIR) sequences as high signal intensity; and inflammation or fibrosis on late gadolinium enhancement sequences again as high signal intensity (Fig. 1) [2, 13, 14]. Histologically, pericardial late gadolinium enhancement correlates with fibroblastic proliferation, neovascularization and chronic inflammation and granulation tissue [15]. Some studies have reported moderate sensitivity (63–68%) and high specificity (up to 100%) of the T2-STIR sequence for acute pericarditis, however this is significantly lower in practice, as elevated signal can also be seen with pericardial effusion or MRI artefact [16, 17]. The delayed enhancement sequence has been reported to have moderate to high 65–100% sensitivity and high specificity 99–100% for pericarditis, however again in practice this is lower with pericardial fat, pleuritis and artefact potentially interfering with scan interpretation, and fat saturated pulses added to delayed enhancement sequences are recommended to improve the positive predictive value of pericardial enhancement [14, 16, 17, 18]. MRI can also evaluate for concomitant myocardial involvement and inflammation (myocarditis) where they be left ventricular dysfunction and regional wall motion abnormalities on cine sequences; increased myocardial signal intensity on T2-STIR or elevated T2-mapping values implying myocardial edema; increased myocardial signal intensity on delayed gadolinium enhancement sequences or elevated T1-mapping values consistent with myocardial inflammation and fibrosis; and along with early gadolinium enhancement suggesting hyperemia [19]. As such MRI is strongly recommended in the initial diagnosis of pericarditis with a complimentary role to clinical, inflammatory biomarkers and echocardiography assessment, especially if the diagnosis remains uncertain after the other tests [1, 2, 10].

Based on MRI and clinical findings, the staging of pericarditis have been proposed which can guide its management [20]. In acute pericarditis, both T2-STIR and delayed gadolinium enhancement are positive for increased signal intensity. The next stage of recurrent pericarditis, delayed enhancement remains positive, while T2-STIR may be positive or negative. In the chronic pericarditis phase, T2-STIR becomes negative while delayed enhancement remains positive. Finally in the burnt out phase, both T2-STIR and delayed enhancement of the pericardium are typically negative. Whereas in the first 2 to 3 phases anti-inflammatory therapies are the cornerstones to therapy, in the refractory or burnt out phase, diuresis for symptoms is often required and pericardiectomy surgery may be considered [10]. Each of these 4 phases may correspond to the transient, subacute or effusive constrictive, chronic and calcific stages of constrictive physiology [20]. Techniques for pericardial enhancement quantitation and semi-quantitation have been proposed with reasonably high intra and inter-observer agreement, and some studies have demonstrated their prognostic value, for example higher grade corresponding to higher rate of treatment failure with anti-inflammatory therapies, higher risk of pericarditis recurrence, but also higher likelihood of constrictive physiology improvement as discussed in a later section [21, 22]. Based on all of these techniques, MRI is a valuable tool in the surveillance of pericarditis to assess treatment response to help guide weaning or identifying the need for additional anti-inflammatory agents.

## 4. Pericardial Effusion and Tamponade

### 4.1 Echocardiography

Pericardial effusion arises from increased production and/or decreased resorption of pericardial fluid leading to abnormal accumulation in the space between the visceral and parietal pericardium [1]. Echocardiography remains the first-line and preferred imaging modality to evaluate for pericardial effusion, and the size is generally classified as trivial (effusion only seen in systole), small (<1 cm), moderate (1.0–1.9 cm), large (>2 cm) and very large (>2.5 cm), irrespective of the presence of tamponade [1, 2]. This should be measured when the adjacent chamber is at its largest during the cardiac cycle (end-ventricular diastole for ventricles, end-ventricular systole for atria), and size is graded as trivial if the pericardial effusion can be seen at some points but not others during the cardiac cycle and <1 cm, whereas small means effusion is seen throughout the cardiac cycle [2, 23]. The effusion can be circumferential or localized, therefore careful interrogation is necessary of parasternal, apical and subcostal windows are required, and sometimes transesophageal echocardiography may be required, such as post-cardiac surgery to identify posterior effusion on transgastric short axis views [2]. It is also important to distinguish pericardial effusion from pleural effusion (for example the latter lies posterior while the formal lies anterior to the descending aorta on parasternal long axis images) as well as epicardial fat pad. Depending on the cause or chronicity of the pericardial effusion, the fluid may contain stranding, loculations or even hematoma, which may affect the appropriate therapy and approach to pericardiocentesis [24].

**Fig. 2.**
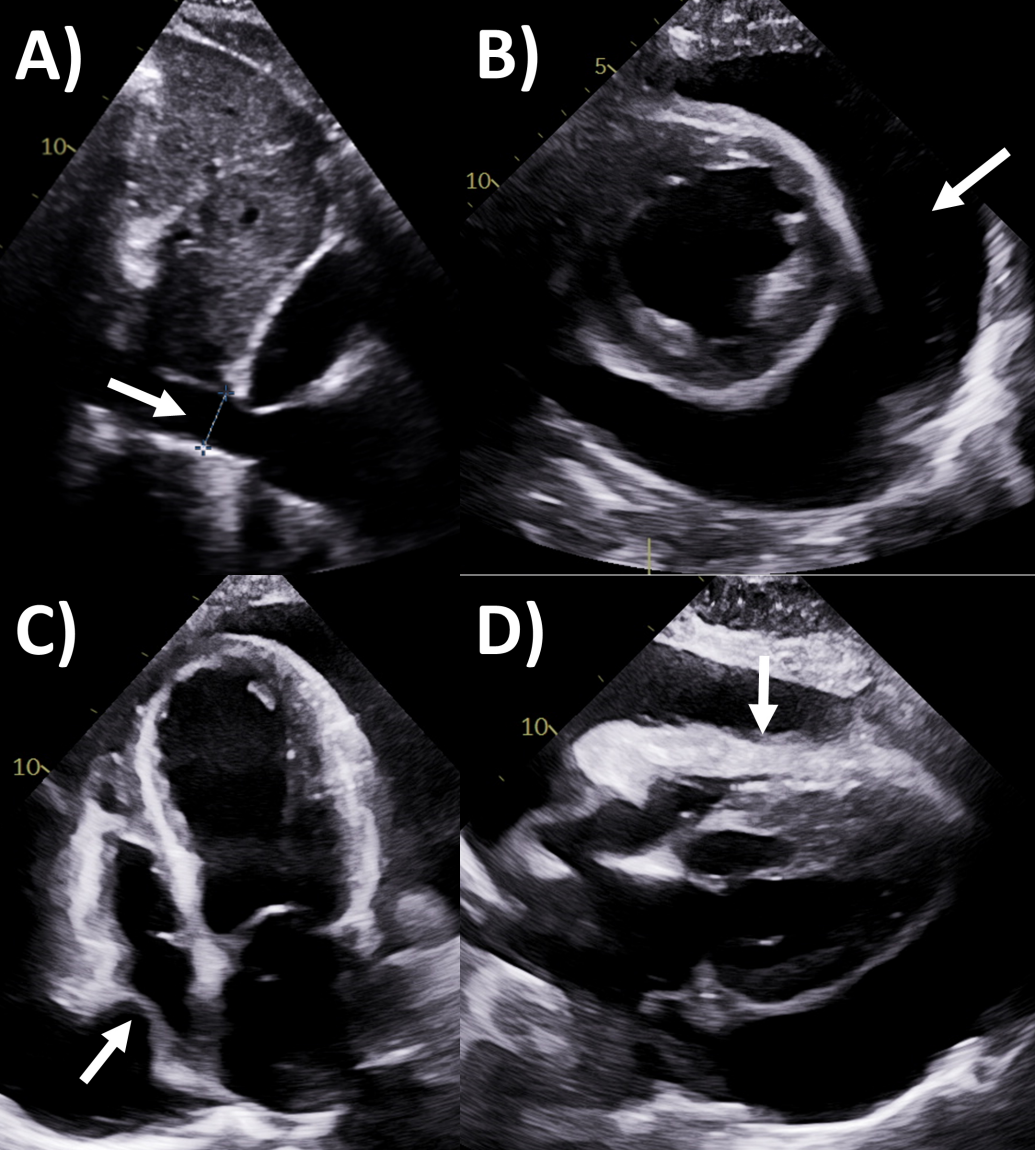
**Echocardiography evaluation of pericardial tamponade**. (A) Dilated inferior vena cava (2.7 cm) with minimal <50% collapse (arrow). (B) Large circumferential pericardial effusion (>3 cm in diastole) on parasternal long axis view (white arrows), with left pleural effusion behind it and the descending aorta (yellow arrow). (C) Right atrial systolic collapse on apical 4-chamber view (arrow). (D) Right and left ventricular diastolic collapse on apical 4-chamber view (white and yellow arrows respectively).

It is critical for cardiology clinicians to be familiar with echocardiographic features that may be seen in pericardial tamponade, despite it ultimately being a clinical diagnosis incorporating signs of hemodynamic compromise [2]. The main echocardiographic criteria according to guidelines (Fig. 2) include dilated inferior vena cava with minimal (<50%) collapse; diastolic collapse with the right ventricle (this sign may be absent in severe pulmonary hypertension with high right ventricular pressure; left heart collapse increases specificity); right atrial collapse (>34% of the duration of cardiac cycle increases specificity); and large pericardial effusion with swinging heart [1, 2, 25, 26, 27]. Other supporting echocardiographic findings for tamponade include significant respirophasic variation of inflows (mitral E wave >30%, tricuspid E wave >60%); diastolic filling blunting and increased expiratory flow reversals of hepatic vein, E/A ratio often <1.0, and large or rapidly enlarged effusion. Tamponade may also be present for loculated effusions or less than large effusion sizes, especially with rapid effusion accumulation [2].

Effusive-constrictive pericarditis is an increasingly recognized entity, where both pathological pericardial features (including tamponade) are concurrently present [2]. There is usually a significant pericardial effusion to start with, and the fluid can be organized, echogenic or loculated by echocardiography [2, 28]. The hallmark feature is that after pericardiocentesis is performed to remove the fluid, there is persistently elevated right atrial pressure, right and left ventricular end-diastolic pressures (with dip and plateau waveform) and prior echocardiography characteristics of constriction such as respirophasic septal shift emerge [2, 29].

### 4.2 Computed Tomography

CT is not commonly used to assess pericardial effusion, usually when incompletely seen on echocardiography or for pre- or post-procedural evaluation [2]. More commonly, the CT chest is performed for another reason, and the pericardial effusion is incidentally found [30]. The main advantages of CT would be to in some cases better visualized the full extent of the pericardial effusion, to characterize the presence of loculations, distinguishing effusion from fat, cyst, hematomas, and masses [2, 31]. By Hounsfield units, fat is usually below 0 (negative), transudative fluid 0–10, exudative fluid 20–60 and blood >60, although there can be some overlap [32].

### 4.3 Magnetic Resonance Imaging

Cardiac MRI is also rarely used to evaluate pericardial effusion and should not be used in unstable patients with suspected tamponade, however both might be incidentally identified when MRI is performed for other indications [33]. As part of pericardial inflammation or constriction assessment, the presence and extent of effusion is also examined. Just like CT, MRI can separate pericardial effusion from cysts, fat and other masses [2, 31]. Pericardial effusion typically has elevated T2-signal but low signal on delayed enhancement sequences, although exudates and blood with higher protein and cell contents increase T1 and reduces T2 signal relative to transudates [34]. MRI findings are tamponade physiology features can are similar to echocardiography such as right ventricular diastolic and/or right atrial systolic collapse, plethoric inferior vena cava and large effusion with swinging heart, generally seen in cine bright-blood sequences imaging [33].

## 5. Pericardial Constriction

### 5.1 Echocardiography

**Fig. 3.**
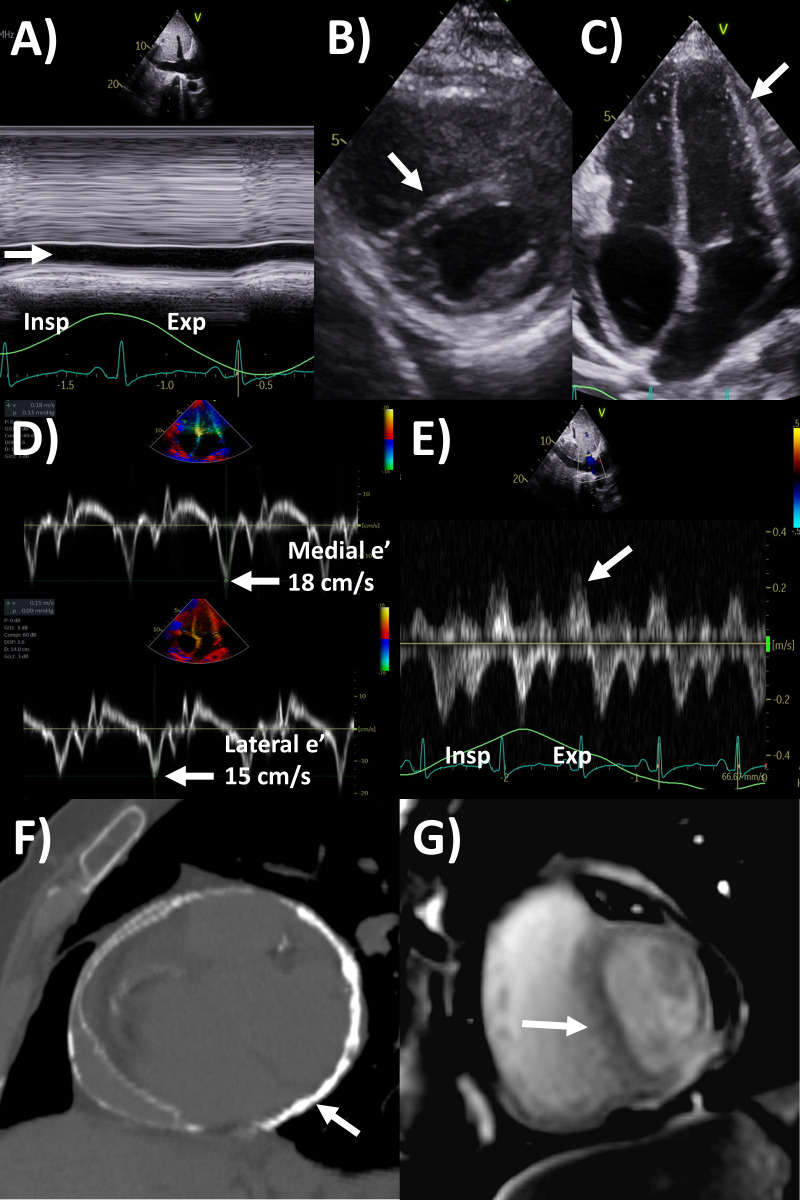
**Multi-modality imaging evaluation of constrictive pericarditis. **Echocardiography (A) dilated inferior vena cava (2.3 cm) with minimal <50% collapse (arrow); (B) respirophasic interventricular septal shift (arrow, insp, inspiration; exp, expiration) on parasternal short axis view; (C) conical deformity of the ventricles (arrow) on apical 4-chamber view, (D) annulus reversus with higher mitral annular medial than lateral e’ (18 versus 15 cm/s, arrows); and (E) increased expiratory end-diastolic reverse velocity/forward velocity at 0.9 of hepatic vein (arrow). Computed tomography (F) extensive pericardial calcifications (arrow). Magnetic resonance imaging (G) free-breathing cine sequence respirophasic interventricular septal shift on short axis view (arrow).

Pericardial constriction is a feared chronic complication of pericardial disease, occurring in approximately 1–2% of pericarditis patients, sharing the same etiology but often challenging to diagnose [1, 35]. Echocardiography is also the first-line and often self-sufficient modality for evaluation of constrictive physiology [2, 36]. The Mayo Clinic Criteria being the recommended algorithm for diagnosing this entity that is frequently challenging to distinguish from restriction or diastolic dysfunction, with several key findings shown in (Fig. 3) [36, 37, 38]. The first part of the algorithm has the dual criteria of mitral inflow E/A ratio >0.8 and dilated inferior vena cava, with the absence of either meaning that constriction or restriction is unlikely. Secondly, respirophasic ventricular septal motion abnormality shift is expected to be present, and cardiac catheterization or further imaging may be considered if this was absent but there are ongoing concerns for constriction. The next key parameter is the mitral medial e’ velocity by tissue Doppler, where >8 cm/s supports constriction diagnosis, <6 cm/s supports restrictive cardiomyopathy, and 6–8 cm/s suggests mixed constriction and restriction heart disease. Furthermore, the presence of annulus reversus (mitral annular lateral e’ being less than medial e’) and/or hepatic vein end-diastolic and expiratory reversal divided by forward flow velocity ≥0.8 are both strongly supportive of constriction.

Of note, in the Mayo Clinic Criteria derivation study, the presence of respirophasic ventricular septal shift and either medial e’ velocity ≥9 cm/s or hepatic vein ratio in expiration ≥0.79 provided the highest diagnostic accuracy for constrictive physiology, with sensitivity 85%, specificity 91%, positive predictive value 97% and negative predictive value 65% [37]. The criteria was externally validated in a cohort study by Cleveland Clinic, with medial e’ velocity being the most important parameter, along with respirophasic septal shift and hepatic vein reversal ratio [39]. Other supportive echocardiography findings for constriction include abnormal pericardial thickening and calcification, tethering of the left or right ventricular free wall or apex to the pericardium or conical deformity, significant respirophasic variation in mitral or tricuspid inflows at least 25 and 40% respectively or that of pulmonary vein diastolic flow, and lower ratio of lateral to septal wall strain [2, 40, 41]. On the other hand, findings supporting restrictive cardiomyopathy include those associated with severe diastolic dysfunction, such as deceleration time <150 ms, isovolumetric relaxation time <50 ms, E/e’ ratio >15 and left atrial volume indexed >48 mL/$m^{2}$ [36].

Transient constriction is increasingly recognized in a subset of ~17% of patients with constrictive physiology, where rather than being chronic it typically resolves within 3–6 months [35, 42]. The underlying pathophysiology is mainly persistent pericardial inflammation rather than fibrosis and calcification seen [43]. Identification of this condition allows timely initiation or continuation of ant-inflammatories to potentially prevent progression to constrictive pericarditis. Not only will there be echocardiography and MRI signs of constrictive physiology, but concomitant pericardial inflammation on MRI (as discussed before, with 86% sensitivity and 80% specificity for this entity) would be observed, whereas pericardial calcification is less often seen on CT [44]. In this sense, inflammation actually portends higher chance of transient constriction and better prognosis.

### 5.2 Computed Tomography

Cardiac CT may assist with diagnosis of pericardial constriction if inconclusive by echocardiography alone [2]. Supportive findings include abnormal thickening 4 mm or more, pericardial calcifications (where CT is the best modality to assess this, although only 25–50% of constriction patients with calcifications), dilated inferior vena cava and atria, conical deformity of the ventricle(s), and extracardiac findings such as pleural effusion, ascites and hepatosplenomegaly [2, 45, 46, 47]. Pericardial calcification often has an irregular distribution, such as preferentially affecting the basal anterolateral left ventricle, right ventricular outflow tract, and adjacent to the mitral and tricuspid annulus [48]. If cine images of the cardiac chambers over one cardiac cycle are performed using retrospective ECG-gating, then respirophasic septal shift and wall tethering may be observed [2]. Perhaps just as valuable is CT’s role in the evaluation of the thoracic anatomy or extracardiac pathologies as part of pre-operative evaluation for pericardiectomy or other cardiothoracic surgeries, such as location of pericardial and aortic calcifications, and cardiovascular structures relative to the sternum which are especially important in redo cardiac surgery [49].

### 5.3 Magnetic Resonance Imaging

MRI is actually a valuable second-line but under-utilized imaging tool for evaluating constrictive pericarditis [2]. Standard cine imaging with steady state free precession or gradient echo sequences not only assess chamber size and function, but also typical constriction findings such as abnormal interventricular septal motion, wall tethering, conical ventricular deformities, and dilated inferior vena cava, while free breathing sequences allows assessment for respirophasic septal shift (Fig. 3) [2, 50]. Pericardial thickness often increased in constrictive pericarditis can be assessed by these bright-blood sequences or black-blood spin echo sequences, as well as dilated inferior vena cava. Quantitative measures include lower short-axis cardiac area at end-inspiration/end-expiration, and higher relative atrial volume index ratio (left versus right) to be present in constrictive pericarditis [51, 52]. Acquiring phase-contrast sequences real-time over 10 seconds with free breathing an detect mitral and tricuspid inflow, with >25% and >45% respiratory variation respectively suggesting constriction [53]. Concurrent MRI findings of pericardial effusion and/or inflammation described in earlier sections must be evaluated [15]. The presence of pericardial inflammation may suggest the disease being earlier in the disease course and higher chance of transient constriction, and is associated with response and improvement to anti-inflammatory therapy, while its absence may push patient towards needing diuresis and potentially pericardiectomy surgery [10, 13]. How well MRI identifies pericardial constriction was evaluated in a study at Cleveland Clinic, which found the combination of relative interventricular septal excursion and pericardial thickness on MRI having the highest discriminative value (c-statistic 0.98, sensitivity 100% and specificity 90%) [54]. Another controlled study found pericardial thickness ≥2 mm, right ventricular end-diastolic volume ≤133 mL and septal flattening by MRI to best discriminate constrictive physiology, all with c-statistics exceeding 0.90 [55].

## 6. Pericardial Masses

### 6.1 Echocardiography

Although echocardiography can identify the presence of pericardial masses, the information it provides can be limited in terms of tissue characterization as well as the extent and spread of the mass. However, some typical tumor features are as follows: haemangioma are usually hyperechoic with septae; lymphangiomas appear heterogenous and hypoechoic with septae, lipomas are usually circumscribed and echogenic; teratomas are usually heterogeneous with both cystic and calcific echogenic areas; lymphoma are usually hypoechoic with effusion; while mesothelioma is usually associated with pericardial thickening and effusion [31, 56]. For pericardial cysts, a hypoechoic space next to the heart chamber border most commonly the right atrium is seen on echocardiography, with lack of blood flow within demonstrated by Doppler or intravenous contrast [31, 57]. Pericardial cyst and diverticula differ where the former has a constant size with altering shape while the other changes in both size and shape with body posture and breathing. Echocardiography may provide guidance in aspiration and drainage of pericardial cysts as well [58].

### 6.2 Computed Tomography

**Fig. 4.**
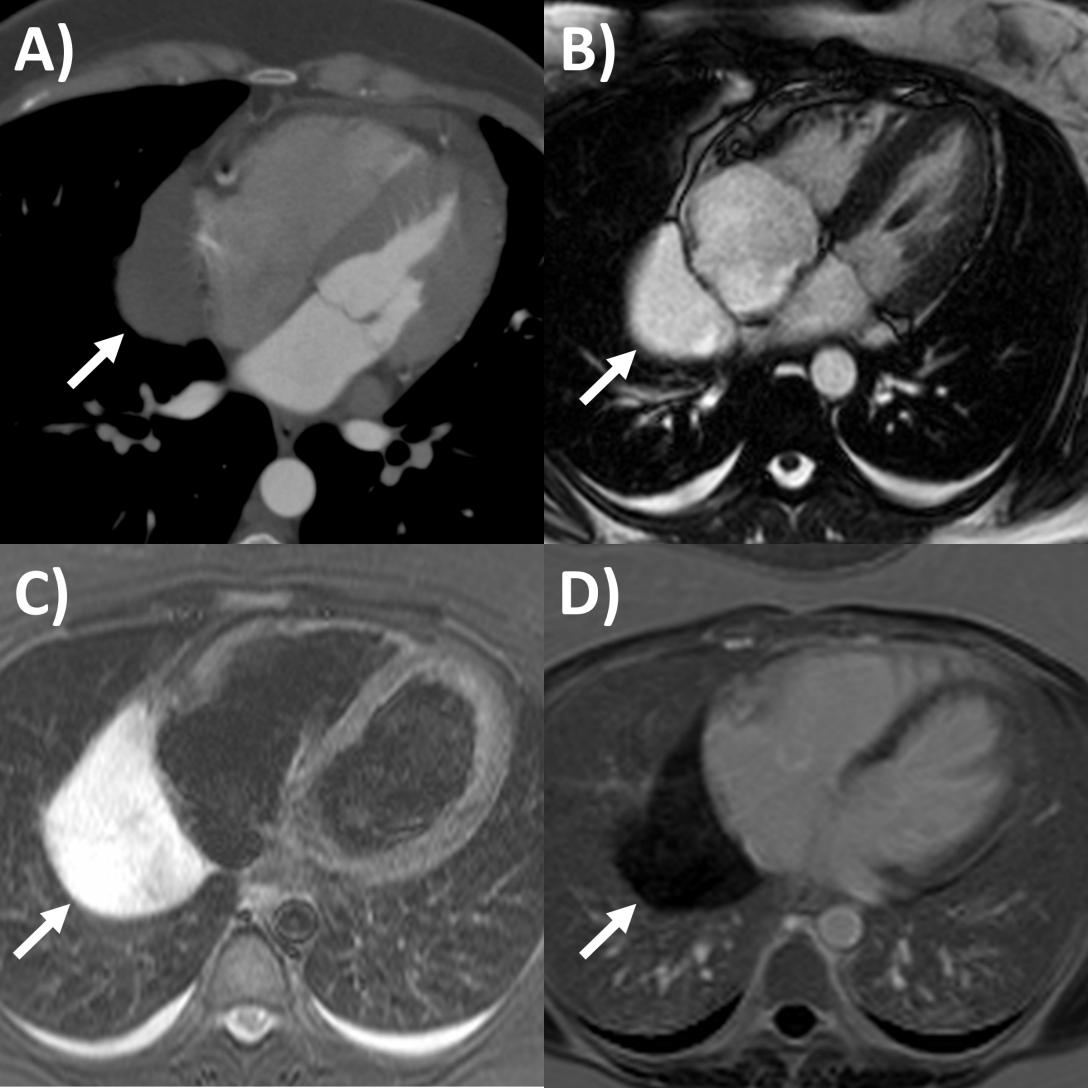
**Multi-modality imaging tissue characterization of pericardial cyst (arrows in all panels) adjacent to the right atrium**. (A) Computed tomography axial slice, cyst was 10 Hounsfield units. (B) Magnetic resonance imaging (MRI) steady-state free precession bright blood sequence axial slice, cyst has increased signal. (C) MRI T2-short tau inversion recovery sequence, cyst has high signal. (D) MRI late gadolinium enhancement sequence axial slice, cyst has low signal.

CT has greater ability for tissue characterization than echocardiography, and is able to assess the extent, local or distant spread of masses, lymph node involvement and many pericardial tumors have associated effusions that are hemorrhagic or exudative [31]. Hemangiomas appear heterogenous with contrast enhancement; lymphangiomas are heterogenous with low attenuation and septae; lipoma have low fat-level attenuation that is circumscribed, and sometimes can surround coronary arteries; teratomas usually have contain areas of calcification and fat; lymphoma are hypoattenuating with contrast enhancement; fibromas are homogeneous with no or minimal enhancement given lack of vascularity; sarcomas are broad-based masses which invade adjacent structures; and mesothelioma is seen as diffuse irregular pericardial thickening with effusion [31, 56]. Pericardial cysts are seen as a well-circumscribed homogeneous mass with thin wall on CT, with fluid density, unaffected by intravenous contrast (Fig. 4) [59, 60].

### 6.3 Magnetic Resonance Imaging

MRI’s main advantage amongst imaging modalities is its ability in tissue characterization, and this is no different when applied to pericardial masses. Depending on tumor extension, the pericardium or myocardium may show thickening, or pericardial effusions, the latter often exudative or hemorrhagic with high signal intensity on T1-weighted sequences [31]. On T1-weighted, T2-weighted and gadolinium enhanced sequences, many tumors have low, high and high signal intensities [14, 31, 61, 62, 63]. Hemangiomas generally appear heterogeneous on all sequences, while lipomas have high signal intensity on all sequences, however its signal can be uniquely suppressed on fat-saturation pulse sequences. Fibroma have low vascularity and therefore have low signal intensity on T2-weighted sequence and none to minimal enhancement on gadolinium enhanced sequences. Mesotheliomas appear homogeneous on T1-weighted but have heterogenenous elevated signal on T2-weighte and gadolinium enhanced sequences. Of note, some studies have suggested heterogenous gadolinium uptake to indicate areas of increased lesion nodularity, growth and/or necrosis [64]. Pericardial cysts also appear as a well-circumscribed homogeneous mass with thin wall on MRI, displaying hypointense signal on T1-weighted sequence unless there is an exudative or hemorrhagic component, with hyperintense signal on T2-weighted sequence and no signal on LGE sequence (Fig. 4) [31, 65]. Lastly, pericardial hematomas show hyperintense, heterogeneous and hypointense signal on T1 and T2 weighted sequences in the acute, subacute and chronic stages, and no signal on LGE sequences regardless of timeframe [31].

## 7. Epicardial Fat 

**Fig. 5.**
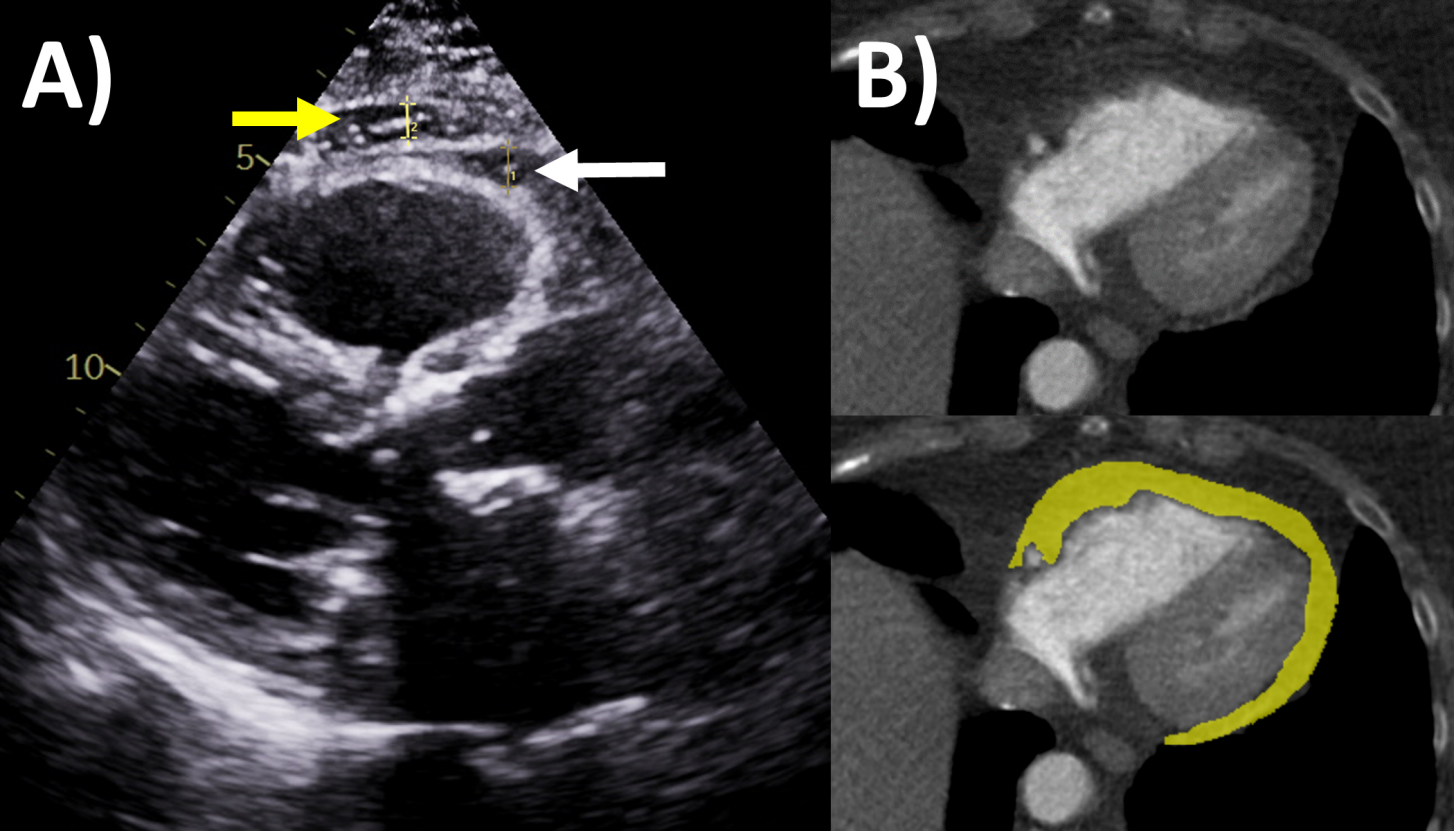
**Epicardial fat evaluation by echocardiography and computed tomography**. (A) Echocardiography: parasternal long axis view, white arrow is epicardial fat measuring 6 mm, yellow arrow is pericardial fat. (B) Computed tomography: yellow area quantifies the epicardial fat on this axial slice though the ventricles.

Epicardial adipose tissue accumulation is often associated with increased body mass index and obesity, and has generated significant clinical interest over the last decade because of its relationships and impact on cardiovascular diseases and events [66, 67]. For example, greater epicardial adipose tissue has been implicated in arrhythmias including atrial fibrillation, ectopy and ventricular arrhythmias, heart failure and coronary heart disease in recent studies [68, 69, 70, 71]. Echocardiography can sometimes measure this as the thickness of echo-lucent space between the visceral pericardium and outer myocardium, typically at the right ventricular free wall in end-systole on parasternal long axis view (Fig. 5A), but the method is limited by inability to measure volumes, assess distribution, and operator dependency [72, 73]. Cardiac CT and MRI have improved ability to accurately measure the epicardial fat thickness, volume and distributions that may not be seen by echocardiography [67, 73]. Epicardial fat appears as fat-level hypoattenuation on CT (Fig. 5B), similar to lipomas. CT has additional capabilities to directly assess peri-coronary epicardial fat, along with coronary artery plaque burden and fat attenuation index, which is a marker of perivascular inflammation often observed adjacent to vulnerable high risk coronary plaque. For MRI, epicardial fat is an important mimic of pericarditis and other pericardial masses, and can be distinguished when its signal becomes low when fat saturation pulses are applied such as on delayed enhancement imaging [14]. Nuclear imaging techniques such as fluorodeoxyglucose-positron emission tomography have also shown promise to assess epicardial fat inflammation associated with coronary stenosis [74]. Assessing epicardial fat should be considered when performing the above imaging modalities in the clinical setting, and further research is needed regarding when dedicated imaging to assess epicardial fat should be performed..

## 8. Congenital Absence of the Pericardium

**Fig. 6.**
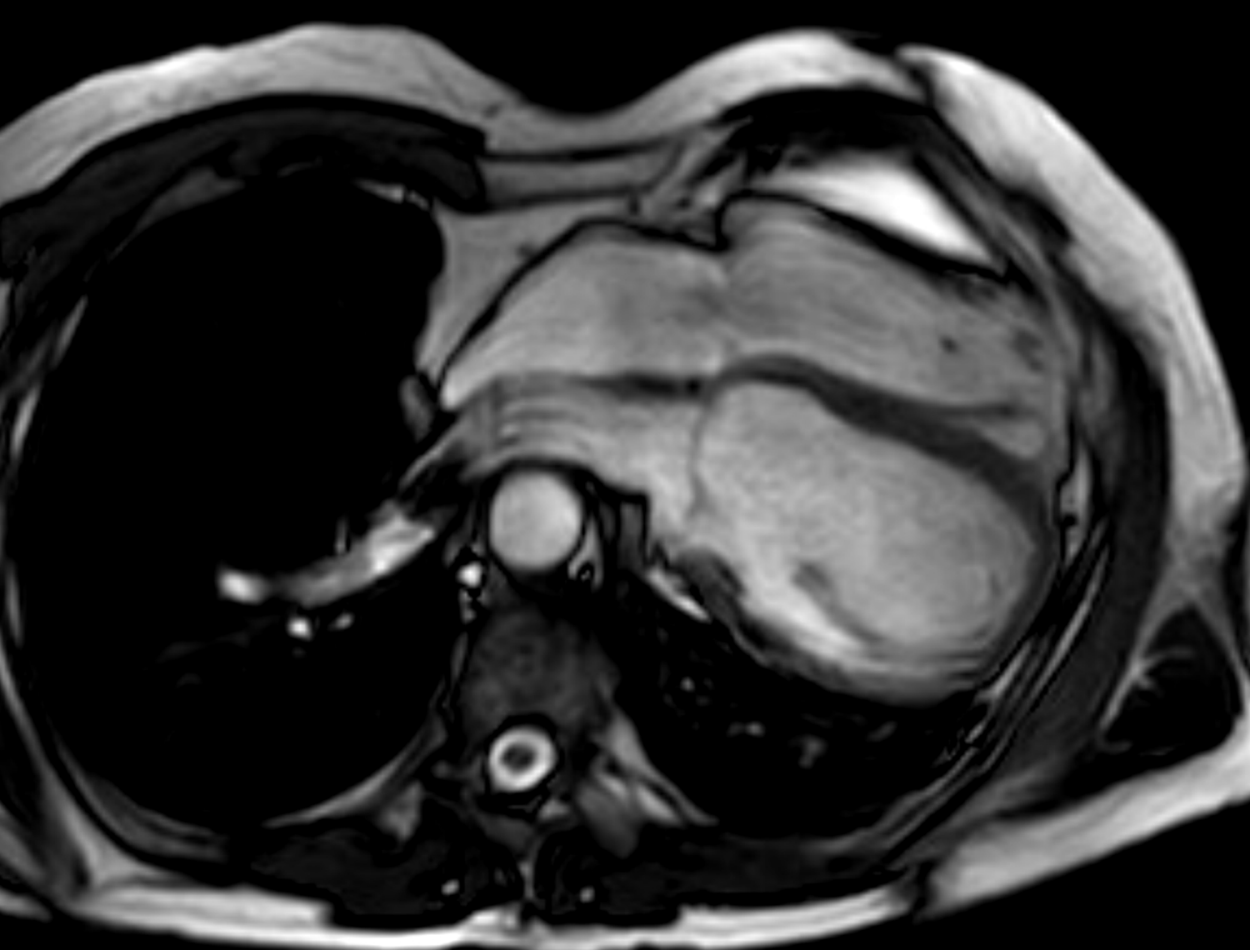
**Congenital absence of the pericardium on magnetic resonance imaging (axial slice steady state free precession bright blood sequence)**.

Congenital absence of the pericardium is a rare anomaly involving partial or complete absence of one or both sides of the pericardium, and complete left-sided absence being the most common [75, 76]. In a minority of patients there are associated other congenital heart diseases such as atrial septal defect and tricuspid atresia [77]. It is often an incidental finding in asymptomatic patients either on imaging (chest or cardiac) or during cardiothoracic surgery, although symptoms may include atypical chest pain, dyspnea, palpitations and dizziness. When echocardiography is performed, typical findings include unusual imaging windows, apparent right ventricular dilation, systolic paradoxical septal motion and excessive cardiac motion [75, 78]. CT and MRI provides direct visualization of its characteristic features in both axial and reconstructed views, such as leftward posterolateral displacement of the apex (levorotation – Fig. 6) and lung tissue interposition in unexpected locations (e.g., between base of heart and diaphragm, between aorta and pulmonay artery) [75]. High-risk imaging findings that may pre-dispose sudden death are left atrial appendage or other chamber herniation and strangulation, left ventricular hinge point or crease, and coronary artery compression or inducible ischemia on stress perfusion [76].

## 9. Conclusions

**Fig. 7.**
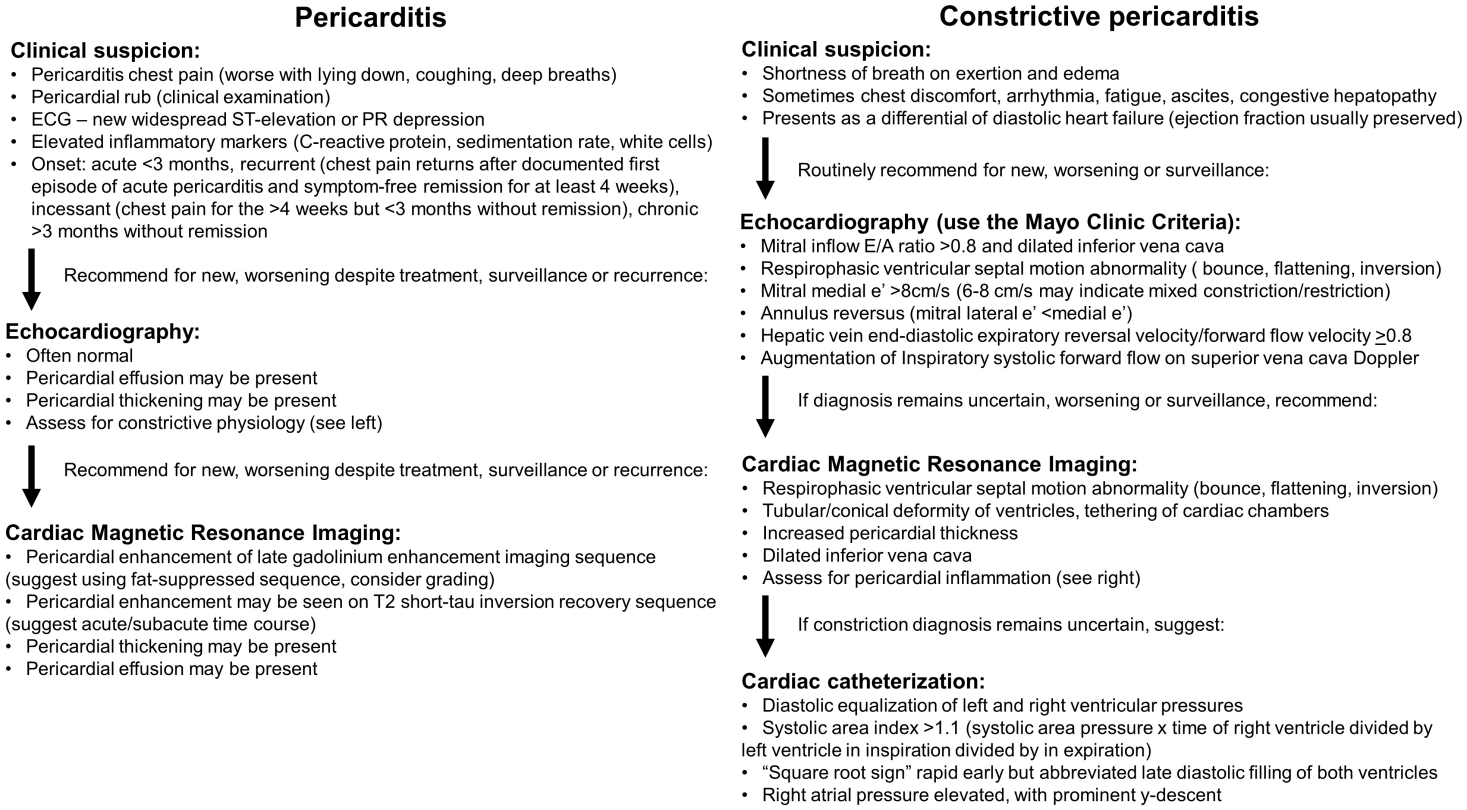
**Proposed multi-modality imaging diagnostic algorithms for constrictive pericarditis and inflammatory pericarditis**.

Multi-modality cardiac imaging is central to the assessment, diagnosis, therapeutic guidance and monitoring of the wide spectrum of pericardial diseases. Each of echocardiography, CT and MRI have unique roles, strengths and limitations to evaluate pericardial anatomy and function (Tables 1,2). These enable them as complimentary tools examine for the presence of one or more of pericardial pathophysiologies such as inflammation, effusion, constriction, masses and congenital anomalies (proposed diagnostic algorithms in Fig. 7 for pericardial inflammation and constriction). Recent advances in multi-modality imaging and novel therapies have led to the rapidly evolving landscape of pericardial disease management, and updated guidelines are urgently needed. Clinicians treating pericardial diseases need to have a sound understanding of these imaging modalities to effectively utilize them to guide management in these often complex patients.
